# The association of dietary vitamin C intake with periodontitis among Korean adults: Results from KNHANES Ⅳ

**DOI:** 10.1371/journal.pone.0177074

**Published:** 2017-05-10

**Authors:** Jung-Hoo Lee, Myung-Seop Shin, Eun-Jeong Kim, Yoo-Been Ahn, Hyun-Duck Kim

**Affiliations:** 1 Department of Preventive and Social Dentistry, School of Dentistry, Seoul National University, Seoul, Korea. 101 Daehak-ro, Jongno-gu, Seoul, Korea; 2 Dental Research Institute, School of Dentistry, Seoul National University, Seoul, Korea; The Ohio State University, UNITED STATES

## Abstract

**Backgrounds:**

The association of dietary vitamin C (vit C) on periodontitis requires more valid evidence from large representative samples to enable sufficient adjustments. This study aimed to evaluate the association between dietary vit C intake and periodontitis after controlling for various confounders in the representative Korean adult population

**Method:**

A total of 10,930 Korean adults (≥19 years) from the fourth Korean National Health and Nutrition Examination Survey data set were included in this cross-sectional study. Periodontitis was defined as community periodontal index score of 3 or 4. Dietary vit C intake was estimated from a 24-hour dietary record, and categorized into adequate and inadequate according to the Korean Estimated Average Requirement value. Potential confounders included age, sex, income, frequency of tooth brushing, use of floss, dental visit, drinking, smoking, diabetes, hypercholesterolemia, hypertension, and obesity. A multivariable logistic regression analysis and stratified analysis were applied.

**Results:**

Those with inadequate dietary vit C intake were more likely by 1.16 times to have periodontitis than those with adequate dietary vit C intake (adjusted odds ratio [aOR] = 1.16, 95% confidence interval = 1.04–1.29). Lowest and middle-low quartile of dietary vit C intake, compared to highest quartile of dietary vit C intake, showed significant association (aOR = 1.28 and 1.22 respectively), which was in a biological-gradient relationship (trend-p <0.05).

**Conclusions:**

Our data showed that inadequate dietary vit C intake was independently associated with periodontitis among Korean adults. Hence, adequate intake of dietary vitamin C could be substantially important on the promotion of periodontal health among Korean adults.

## Introduction

Periodontitis is a specific inflammatory response to periodontal microorganisms which result in the destruction of the periodontal ligament and bone [1]. Periodontitis is a multifactorial disease and the risk factors for periodontitis include age, sex, socioeconomic status [2], and smoking [3]. Moreover, recent Korean data showed that cardiovascular diseases and metabolic syndrome were also related to periodontitis [4–6]. Periodontitis is prevalent among Korean adults, showing the prevalence range of 10.2% to 55.7% across different age groups [7].

The excessive production of reactive oxygen species (ROS) or reduced antioxidant levels has been associated with periodontitis [8,9]. For the scavenging of excessive ROS, many kinds of antioxidants are needed in our body and vitamin C (vit C) is one of important dietary antioxidants which has been associated with periodontal health [10]. Java prospective study showed controversial results: non-fasting serum vit C increased the incidence of periodontitis for three-year follow-up (2002–2005) [11]. However, fasting serum vit C did not increased the incidence of periodontitis for six-year intervention (2005–2011) [12]. Some used serum vit c [13,14,16–19] and some used dietary vit c [15, 20]. Although Clark et al. [21] who reported no association of dietary vit C with periodontitis from a longitudinal study among pregnant women suggested some more longitudinal epidemiological researches, few study could be found.

Moreover, most of studies had limitations such as small sample size [11–14] or lack of adjustment for systemic health-related confounders [16]. Hence, studies with large representative sample and adjustment for potential confounders are needed. Moreover, no study has addressed the association between vit C intake and periodontitis with enough adjustment for potential confounders among Korean adults.

The fourth Korean National Health and Nutrition Examination Survey (KNHANES Ⅳ) has provided information about dietary vit C intake, periodontitis and systemic factors. This study using KNHANES Ⅳ data set aimed to evaluate the association between dietary vit C intake and periodontitis after controlling for age, sex, income, frequency of tooth brushing, use of dental floss, dental visit, alcohol drinking, smoking, diabetes, hypercholesterolemia, hypertension, and obesity in a representative Korean adult population

## Materials and methods

### Study design and participants

KNHANES is representative of the Korean civilian, non-institutionalized population. KNHANES Ⅳ was a cross-sectional survey conducted by the Korea Center for Disease Control and Prevention (KCDCP) from 2007 to 2009. All participants provided a written informed consent for voluntary participation. The three-stage, stratified, clustered sampling method were used and unequal weight, of which the sum was 100%, was given to each individual. The total participants for KNHANES IV were 24,871. The inclusion criteria of this study were: 1) adult aged over 19 years, 2) those with valid periodontal examination data without edentulous status, 3) participants without missing variables. Among a total of 18,406 participants who were aged 19 years or more, 16,279 participants took periodontal examination and were not edentulous, and 14,227 participants had valid nutrition data. Finally, 10,930 participants who had no missing variable joined in the final analysis.

### Assessment of periodontitis

The periodontal status was evaluated by community periodontal index (CPI) developed by World Health Organization (WHO) [22]. Ten index teeth, which are two molars in each posterior sextant, and the upper right and lower left central incisors, were measured for periodontal pocket depth by using a CPI probe (Osung MND, Seoul, Korea) at six sites per tooth (mesio-buccal, mid-buccal, disto-buccal, mesio-lingual, mid-lingual, disto-lingual). Probing was conducted by dentists who had received calibration training. Five CPI scores were recorded: normal gingiva (CPI 0), bleeding on probing (CPI 1), presence of gingival calculus (CPI 2), shallow periodontal pocket of 3.5–5.5mm (CPI 3) and deep periodontal pocket of 5.5 mm or more (CPI 4). Periodontitis was defined as CPI scores of 3 or 4 and participants were classified into two groups: non-periodontitis and periodontitis. The inter-examiner means of the Kappa value for CPI were 0.72 (0.69~0.77) in 2007, and 0.89 (0.55~1.00) in 2008 and 0.75 (0.53~0.94) in 2009 [23].

### Assessment of vitamin C intake

Dietary food intake information was estimated by using a 24-hour dietary record (24-hDR) of the day before examination with interview using questionnaires [24,25]. The daily food intake was calculated and converted into an estimate of daily vit C intake using the standard food nutrient composition database and calculation formula guideline of Korean Nutrition Society (KNS) [26]. The Estimated Average Requirement (EAR), one of the Dietary Reference Intakes (DRI), is defined as the nutrient values estimated to meet the nutrient requirements in 50% of the healthy individuals in certain life stage and sex group, while the other remaining 50% are in inadequate state [27, 28]. The cut-off value of vit C by EAR is 75 mg/day for Korean adults [26]. Whereas, the Recommended Nutrient Intake (RNI) is the daily intake level of nutrients to meet the requirement of nearly all (97–98%) individuals. The cut-off value of vit C by RNI is 100 mg/day for Korean adults [26]. The amount of dietary vit C intake was categorized into adequate and inadequate according to EAR value and quartile value. For the quartile values, dietary vit C intake was classified into the four category; Highest (≥132.22 mg/day), Middle high (80.18 to <132.22 mg/day), Middle low (47.34 to <80.18 mg/day) and Lowest (<47.34 mg/day) in the overall sample.

### Assessment of confounders

Since vitamin C is associated with various confounders including age, sex, education, smoking, alcohol consumption, obesity and physical activity [29], selected potential confounders included socio-demographic factors such as age, sex and income, oral health behaviors such as tooth brushing, use of floss, and dental visit, behavioral factors such as alcohol drinking and lifetime smoking and systemic factors such as diabetes mellitus, hypercholesterolemia, hypertension and obesity. The socio-demographic, oral health behaviors and behavioral factors were surveyed with questionnaires in an interview. The general health-related factors were assessed not only by questionnaires but clinical examination and laboratory procedures.

Age was classified into seven groups: 19 to 29, 30 to 39, 40 to 49, 50 to 59, 60 to 69 and 70 years or more. The monthly house hold income which is adjusted for the family number classified into quartiles groups according to monthly household income: the lowest, middle-low, middle-high, and the highest. Frequency of tooth brushing was classified into three categories: once or no per day, two times per day, and over three times per day. Use of floss was grouped into yes or no. Dental visit were classified into four categories: until the last 6 months, over 6 months to two years, over two years, and unknown. Alcohol drinking frequency in the last one year was categorized into 4 groups: 0–1time per month, 2–4 times per month, 2–3 times per week, 4 or more times per week. Lifetime smoking was categorized into three groups: No, below 5 packs (<100 cigarettes) and 5 packs or more (≥100 cigarettes). Diabetes was classified into three groups: diabetes for fasting blood sugar (FBS) ≥126 mg/dl or diagnosed or medicated for diabetes, pre-diabetes for 100≤FBS<126mg/dl and normal for FBS<100mg/dl. Hypercholesterolemia was classified into two groups: hypercholesterolemia for Total Cholesterol ≥240 mg/dl or on lipid-lowering medication and normal for Total Cholesterol <240 mg/dl. Hypertension was classified into three groups: hypertension for systolic blood pressure (SBP) ≥140mmHg or diastolic blood pressure (DBP) ≥90mmHg or on anti-hypertensive medication, pre-hypertensive for 120≤SBP<140mmHg or 80≤DBP<90mmHg, and normal for SBP<120mmHg and DBP<80mmHg. Obesity was classified into three statuses according to body mass index (BMI): normal for 18.5kg/m^2^ ≤ BMI <25kg/m^2^, underweight for BMI<18.5kg/m^2^, and obese for BMI ≥ 25kg/m^2^.

### Statistical analysis

For the characteristics of the participants by periodontitis status, Chi-square test of complex sample analysis with weight application was performed to estimate the weighted proportions (95% confidence interval [CI]) of the total population sample. Crude associations between independent categorical variables and periodontitis were calculated by un-weighted chi-square test.

Multiple logistic regression analysis of complex sample analysis with applying weight was performed to evaluate the adjusted association between dietary vit C intake (EAR-defined, quartile) and periodontitis. Model 1 was analyzed by EAR value, whereas Model 2 was analyzed by quartile value of dietary vit C intake to confirm whether dietary vit C intake has biological-gradient relationship for periodontitis. Stratified analysis according to age group and sex was performed to reduce the positive bias due to the big sample with over 10,000 participants.

To evaluate the risk groups according to confounding variables, the subsequent stratified analysis among adults aged 30–49 years was performed. In the subsequent stratified analysis, we combined categories (pre-diabetes and diabetes for diabetes stratum, low and low middle for income stratum) to ensure adequate sample size. Moreover, we added the analysis of the association between RNI defined vit C intake and periodontitis among adults aged 30–49 years for more sensitive analysis. We used SPSS package (version 21, IBM, SPSS Inc., Chicago IL.USA) for data analysis.

## Results

### Characteristics of the participants

The prevalence of periodontitis in adults was 32.0% in our data. Periodontitis was more prevalent among elders, males, those with less vit C intake, lower income, frequent alcohol drinkers, heavy smokers, less tooth brushing, non-use of floss, diabetes, hypercholesterolemia, hypertension and obesity (Table 1).

**Table 1:** Characteristics of the participants by periodontitis status (N = 10,930).

Variables	Category	N	Periodontitis*				P value^†^
			No		Yes		
			N	% (95% CI) ^†^	N	% (95% CI) ^†^	
**Total**		10930	7118	68.0 (66.5–69.6)	3812	32.0 (30.4–33.5)	
**Age groups (years)**	19–29	1515	1403	92.7 (90.8–94.2)	112	7.3 (5.8–9.2)	<0.001
	30–39,	2529	2047	79.5 (77.0–81.8)	482	20.5 (18.2–23.0)	
	40–49	2378	1497	59.8 (57.1–62.5)	881	40.2 (37.5–42.9)	
	50–59	1875	945	48.7 (45.6–51.9)	930	51.3 (48.1–54.4)	
	60–69	1614	754	43.8 (40.3–47.5)	860	56.2 (52.5–59.7)	
	≥70	1019	472	44.1 (39.9–48.3)	547	55.9 (51.7–60.1)	
**Sex**	Male	4864	2736	62.0 (60.0–64.0)	2128	38.0 (36.0–40.0)	<0.001
	Female	6066	4382	75.1 (73.4–76.7)	1684	24.9 (23.3–26.6)	
**Monthly household Income**	Lowest	1940	1030	58.3 (54.9–61.6)	910	41.7 (38.4–45.1)	<0.001
	Middle low	2674	1664	65.5 (63.0–68.0)	1010	34.5 (32.0–37.0)	
	Middle high	3093	2085	68.3 (66.1–70.5)	1008	31.7 (29.5–33.9)	
	Highest	3223	2339	73.7 (71.3–75.9)	884	26.3 (24.1–28.7)	
**Frequency of tooth brushing**	Once or no	1365	756	58.3 (54.9–61.6)	609	41.7 (38.4–45.1)	<0.001
	Twice	4590	2846	64.9 (62.8–66.9)	1744	35.1 (33.1–37.2)	
	Over 3 times	4975	3516	73.0(71.2–74.7)	1459	27.0 (25.3–28.8)	
**Use of floss**	No	9532	6000	66.2 (64.5–67.8)	3532	33.8 (32.2–35.5)	<0.001
	Yes	1398	1118	80.1 (77.4–82.6)	280	19.9 (17.4–22.6)	
**Dental visit**	No	342	229	71.1 (64.9–76.7)	113	28.9 (23.3–35.1)	<0.001
	6 months	2999	1929	66.8 (64.5–68.9)	1070	33.2 (31.1–35.5)	
	6 m to 2 years	3867	2592	70.1 (68.0–72.1)	1275	29.9 (27.9–32.0)	
	Over 2 years	3705	2355	66.7 (64.4–68.9)	1350	33.3 (31.1–35.6)	
	Unknown	17	13	74.4 (43.4–91.7)	4	25.6 (8.3–56.6)	
**Alcohol Drinking (time)**	0–1/month	5591	3741	70.1 (68.2–71.9)	1850	29.9 (28.1–31.8)	<0.001
	2–4/month	2713	1881	72.3 (69.9–74.5)	832	27.7 (25.5–30.1)	
	2–3/week	1717	1068	64.9 (62,0–67.6)	649	35.1 (32.4–38.0)	
	≥4/week	909	428	48.2 (43.9–52.4)	481	51.8 (47.6–56.1)	
**Lifetime smoking**	No	6159	4424	75.4 (73.6–77.0)	1735	24.6 (23.0–26.4)	<0.001
	<5pack	309	232	80.3 (74.6–85.0)	77	19.7 (15.0–25.4)	
	≥5packs	4462	2462	59.5 (57.4–61.6)	2000	40.5 (38.4–42.6)	
**Diabetes**^**‡**^	Normal	7981	5598	72.9 (71.3–74.4)	2383	27.1 (25.6–28.7)	<0.001
	Pre-diabetes	1999	1081	56.1 (53.0–59.1)	918	43.9 (40.9–47.0)	
	Diabetes	950	439	45.5 (41.4–49.7)	511	54.5 (50.3–58.6)	
**Hypercholesterolemia**^**§**^	No	9818	6501	69.2 (67.6–70.7)	3317	30.8 (29.3–32.4)	<0.001
	Yes	1112	617	56.6 (52.9–60.3)	495	43.4 (39.7–47.1)	
**Hypertension**^║^	Normal	5341	3948	75.7 (73.9–77.3)	1393	24.3 (22.7–26.1)	<0.001
	Prehypertension	2684	1665	66.1 (63.4–68.6)	1019	33.9 (31.4–36.6)	
	Hypertension	2905	1505	53.1 (50.4–55.9)	1400	46.9 (44.1–49.6)	
**Obesity**^¶^	Normal	7031	4701	70.1 (68.4–71.8)	2330	29.9 (28.2–31.6)	<0.001
	Underweight	512	386	78.0 (73.7–81.8)	126	22.0 (18.2–26.3)	
	Obese	3387	2031	62.3 (59.9–64.6)	1356	37.7 (35.4–40.1)	
**Dietary vitamin C (EAR)**^**#**^	Adequate	5869	3895	68.5 (66.7–70.4)	1974	31.5 (29.6–33.3)	<0.05
	Inadequate	5061	3223	67.4 (65.5–69.3)	1838	32.6 (30.7–34.5)	
Quartile diet vitamin C^**^	Highest	2732	1847	70.2 (67.8–72.4)	885	29.8 (27.6–32.2)	<0.05
	Middle high	2733	1792	67.6 (65.2–70.0)	941	32.4 (30.0–34.8)	
	Middle low	2733	1763	66.8 (64.4–69.1)	970	33.2 (30.9–35.6)	
	Lowest	2732	1716	67.5 (65.1–69.8)	1016	32.5 (30.2–34.9)	

*Periodontitis was defined as community periodontal index 3–4.

^†^Weighted percent, 95% Confidence Interval (CI), and *p*-value obtained by Chi-square test.

^**‡**^Diabetes: Normal; (FBS) <100mg/dl, pre-diabetes; 100≤FBS≤125mg/dl, Diabetes; (FBS) ≥126mg/dl or on anti-diabetic medicine or insulin injection or on diagnose by doctor.

^**§**^Hypercholesterolemia: Fasting Total Cholesterol (FTC) ≥ 240 mg/dl or anti-cholesterol drug.

^║^Hypertension: Normal; systolic blood pressure (SBP) <120mmHg and diastolic blood pressure (DBP) <80mmHg, Pre-hypertension; 120≤ SBP <140mmHg or: 80≤ DBP <90mmHg, Hypertension; SBP ≥140mmHg, or DBP ≥90mmHg or anti-hypertensive medicine.

^¶^Obesity; Normal; Body Mass Index (BMI) 18.5 to <25, Underweight: BMI <18.5, and Obesity: BMI ≥25.0 kg/m^2^.

^#^Dietary vitamin C; Adequate: ≥75mg/day and Inadequate: <75mg/day by estimated average requirement (EAR).

**Quartile diet vit C; Highest (≥132.22 mg/day), middle high (80.18 to <132.22 mg/day), middle low (47.34 to <80.18 mg/day) and Lowest (<47.34 mg/day).

### Association between dietary vit C intake and periodontitis in overall sample

EAR-defined dietary vit C intake was significantly associated with periodontitis after controlling for all the possible confounders (Table 2). Those with inadequate intake of dietary vit C were more likely to have periodontitis by 1.16 times than those with adequate intake of dietary vit C. Especially, quartile dietary vit C intake was also associated with periodontitis. Lowest and middle-low quartile of dietary vit C intake, compared to highest quartile of dietary vit C intake, showed significant association of 1.28 times and 1.22 times respectively, which was in a biological-gradient relationship (trend-p <0.05). Our data showed that age, sex and income, use of floss, and dental visit, alcohol drinking and lifetime smoking and diabetes mellitus were also significantly associated with periodontitis. Generally, Koreans visit dental office for dental treatment rather than regular check-up. Especially, those who visit the dental office were more likely to have periodontitis by 1.4–1.5 times than those who did not visit the dental office.

**Table 2:** Adjusted association between dietary vitamin C and periodontitis (N = 10,930).

Variable	Subgroups	N	Odds ratio* (95% Confidence Interval)	
			Model 1	Model 2
Dietary vitamin C^†^	Adequate	5869	1	
	Inadequate	5061	**1.161 (1.042–1.294)**	
Quartile values^**‡**^	Highest	2732		1
	Middle high	2733		1.120 (0.973–1.289)
	Middle low	2733		**1.225 (1.054–1.423)**
	Lowest	2732		**1.282 (1.099–1.497)**
				(Trend-p = 0.012)
Age groups	19–29	1515	1	1
	30–39	2529	**3.321 (2.518–4.379)**	**3.325 (2.520–4.388)**
	40–49	2378	**8.519 (6.546–11.088)**	**8.539 (6.561–11.114)**
	50–59	1875	**12.581 (9.466–16.720)**	**12.615 (9.491–16.767)**
	60–69	1614	**13.329 (9.825–18.081)**	**13.334 (9.822–18.101)**
	70	1019	**12.216 (8.869–16.827)**	**12.163 (8.818–16.776)**
Sex	Male	4864	1	1
	Female	6066	**0.754 (0.644–0.884)**	**0.750 (0.640–0.880)**
Monthly house Income	Low	1940	1	1
	Middle-low	2674	0.989 (0.832–1.177)	0.991 (0.833–1.179)
	Middle-high	3093	0.954 (0.795–1.145)	0.958 (0.799–1.149)
	High	3223	**0.713 (0.584–0.871)**	**0.717 (0.587–0.876)**
Tooth brushing	Once or no/day	1365	1	1
	Twice /day	4590	1.015 (0.863–1.194)	1.017 (0.864–1.196)
	Over 3 times/day	4975	0.908 (0.767–1.074)	0.910 (0.769–1.076)
Use of floss	No	9532	1	1
	Yes	1398	**0.694 (0.583–0.827)**	**0.696 (0.584–0.830)**
Dental visit	No visit	342	1	1
	Last 6 months	2999	**1.517 (1.101–2.091)**	**1.522 (1.105–2.097)**
	Last 6 months to 2 years	3867	**1.408 (1.022–1.940)**	**1.413 (1.025–1.947)**
	Over last 2 years	3705	**1.485 (1.068–2.066)**	**1.489 (1.071–2.071)**
	Unknown	17	1.607 (0.295–8.742)	1.606 (0.294–8.778)
Alcohol drinking	0–1/month	5591	1	1
	2–4/month	2713	1.024 (0.894–1.172)	1.022 (0.893–1.171)
	2–3/week	1717	1.015 (0.866–1.189)	1.007 (0.859–1.181)
	≥4/week	909	**1.264 (1.033–1.548)**	**1.262 (1.032–1.544)**
Lifetime Smoking	No	6159	1	1
	<5packs	309	0.899 (0.613–1.318)	0.896 (0.611–1.312)
	≥5packs	4462	**1.643 (1.400–1.928)**	**1.639 (1.396–1.924)**
Diabetes	Normal	7981	1	1
	Pre-diabetes	1999	**1.183 (1.037–1.350)**	**1.184 (1.039–1.351)**
	Diabetes	950	**1.359 (1.126–1.639)**	**1.359 (1.126–1.639)**
Hypercholesterolemia	No	9818	1	1
	Yes	1112	1.044 (0.893–1.220)	1.045 (0.895–1.221)
Hypertension	Normal	5341	1	1
	Prehypertension	2684	1.047 (0.903–1.214)	1.047 (0.903–1.214)
	Hypertension	2905	1.036 (0.887–1.210)	1.037 (0.888–1.211)
Obesity	Normal	7031	1	1
	Underweight	512	1.159 (0.886–1.516)	1.153 (0.881–1.508)
	Obese	3387	1.096 (0.969–1.240)	1.097 (0.970–1.241)

Periodontitis was defined as community periodontal index 3–4.

*Odds ratios in Model 1 and 2 are adjusted for age, sex, income, tooth brushing, use of floss, dental visit, alcohol drinking, smoking, diabetes, hypercholesterolemia, hypertension, and obesity.

^†^ Dietary vitamin C; Adequate: ≥75mg/day and Inadequate: <75mg/day by Estimated Average Requirement value.

^**‡**^Quartile value; Highest (≥132.22 mg/day), middle high (80.18 to <132.22 mg/day), middle low (47.34 to <80.18 mg/day) and Lowest (<47.34 mg/day).

Bold denotes statistical significance at *p*〈0.05

### Age and sex stratified association between EAR-defined dietary vit C and periodontitis

In the age stratified analysis, the association of inadequate intake of vit-C was highlighted among those aged 30–39 years (adjusted Odds Ratio [aOR] = 1.26, 95% confidence interval [CI] = 1.004–1.58) and 40–49 years (aOR = 1.36, 95% CI = 1.10–1.67), while those in other age groups were not statistically significant (Table 3). In terms of sex, the association was highlighted in females (aOR = 1.30, 95% CI = 1.12–1.51). In terms of age groups and sex, the link was highlighted among males aged 40–49 year group and females aged 30–39 and 40–49 years groups (Table 3). Of them, the link was the highest among females aged 30–39 years group (aOR = 1.62, 95% CI = 1.15–2.25).

**Table 3:** Age and sex stratified association of dietary vitamin C intake (inadequate against adequate) with periodontitis (N = 10,930).

Stratum	Subgroup	N	Odds ratio* (95% confidence interval)
Age	19–29 years	1515	1.091 (0.681–1.749)
	**30–39 years**	**2529**	**1.260 (1.004–1.581)**
	**40–49 years**	**2378**	**1.361 (1.105–1.675)**
	50–59 years	1875	1.048 (0.825–1.331)
	60–69 years	1614	1.034 (0.797–1.342)
	≥70 years	1019	0.922 (0.668–1.272)
Sex	Male	4864	1.054 (0.909–1.222)
	**Female**	**6066**	**1.305 (1.123–1.516)**
Age and sex			
Male	19–29 years	641	0.995 (0.514–1.925)
	30–39 years	969	1.078 (0.784–1.483)
	**40–49 years**	**998**	**1.408 (1.049–1.888)**
	50–59 years	856	0.872 (0.626–1.216)
	60–69 years	835	0.921 (0.648–1.309)
	≥70 years	565	0.938 (0.607–1.449)
Female	19–29 years	874	1.265 (0.689–2.323)
	**30–39 years**	**1560**	**1.615 (1.158–2.253)**
	**40–49 years**	**1380**	**1.303 (1.001–1.694)**
	50–59 years	1019	1.271 (0.931–1.735)
	60–69 years	779	1.174 (0.827–1.666)
	≥70 years	454	0.964 (0.593–1.568)

Periodontitis was defined as community periodontal index 3–4.

* Odds ratio of dietary vitamin C defined by EAR (Inadequate: [< 75mg/day] against the reference, Adequate: [≥75mg/day]) was adjusted for age, sex, income, frequency of tooth brushing, use of floss, dental visit, alcohol drinking, smoking, diabetes, hypercholesterolemia, hypertension, and obesity except for stratum

Bold denotes statistical significance at *p*〈0.05

#### Sensitivity of the link between dietary vit C and periodontitis among Korean adults aged 30 to 49 years

**Fig 1:**
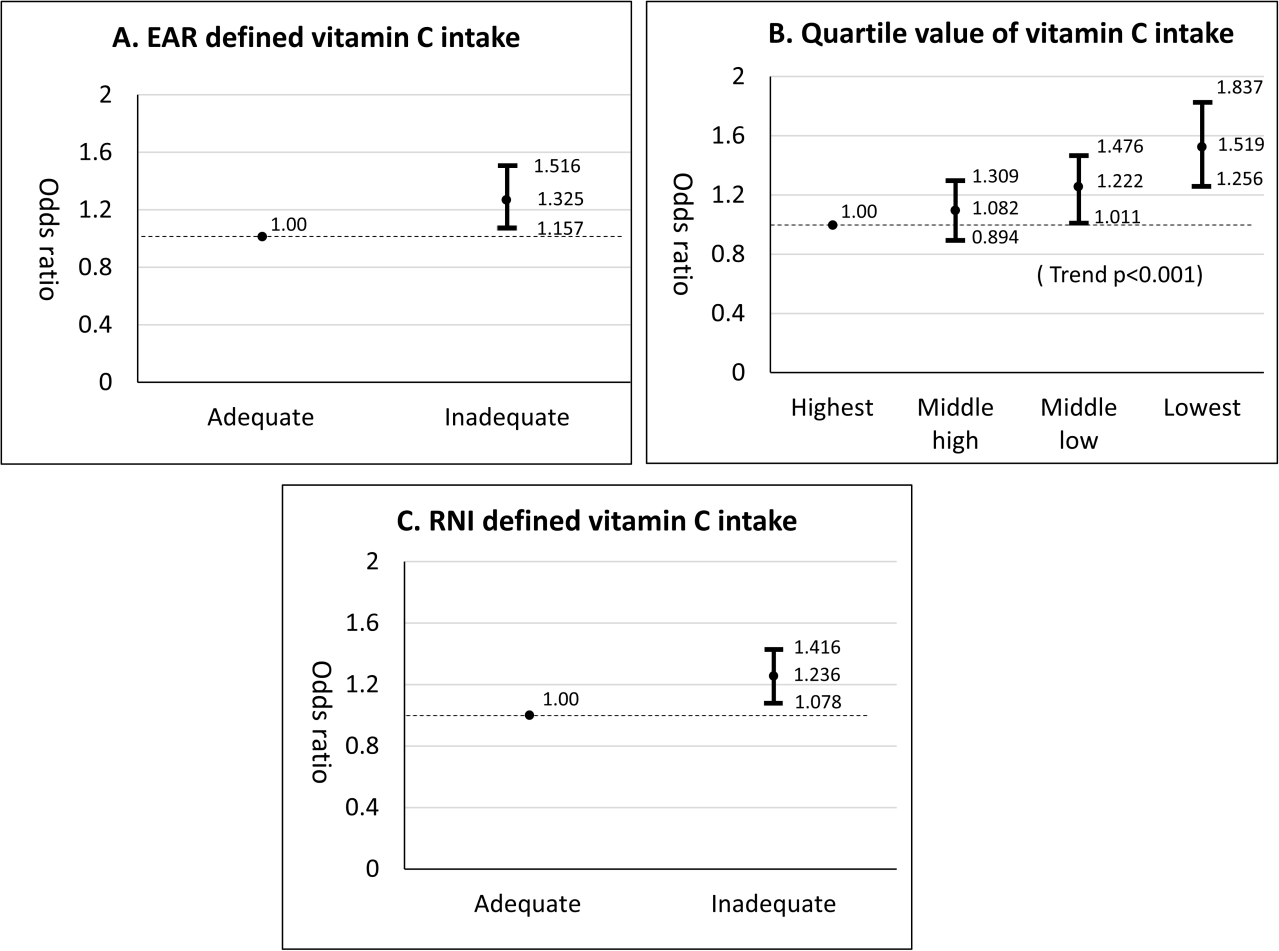
Sensitivity of association between dietary vitamin C intake and periodontitis among Korean adults aged 30 to 49 years (N = 4907). A. EAR defined vit C intake; B. Quartile vit C intake C.RNI defined vit C intake. Odds ratios were adjusted for age, sex, income, frequency of tooth brushing, use of floss, dental visit, drinking, smoking, diabetes, hypercholesterolemia, hypertension, and obesity. Dots indicate odds ratios, bars indicate 95% confidence intervals and the horizontal dotted line is the reference (odds ratio = 1).

EAR-defined dietary vit C intake was associated with periodontitis among Korean adults aged 30 to 49 years (aOR = 1.33, 95% CI = 1.16–1.52) (Fig 1), which was confirmed by two other analyses, quartile dietary vit C intake and RNI-defined dietary vit C intake analyses. The association between quartile dietary vit C intake and periodontitis also showed a biological-gradient relationship (trend-p <0.05). Moreover, RNI-defined dietary vit C intake also showed a significant association with periodontitis (aOR = 1.24, 95% CI = 1.08–1.42).

#### Socio-behavioral-systemic factors stratified association between dietary vitamin C intake (inadequate against adequate) and periodontitis among Korean adults aged 30 to 49 years

Among adults aged 30–49 years, the link between EAR defined vit C intake (inadequate against adequate) and periodontitis was highlighted among those with middle or low income, drinking frequency of two to four times per month, no smoking, prediabetes or diabetes, prehypertension or hypertension (Table 4). Of them, the link was the highest among those with pre-diabetes or diabetes (aOR = 1.68, 95% CI = 1.26–2.23).

**Table 4:** Socio-behavioral-systemic factors stratified association between dietary vitamin C intake (inadequate against adequate) and periodontitis among adults aged 30–49 years (n = 4907).

Subgroup	N	Odds ratio* (95% confidence interval)
Monthly house Income		
<50%	1381	1.339 (1.054–1.701)
50–75%	1744	1.376 (1.094–1.731)
>75%	1782	1.293 (1.020–1.640)
Drinking (times/week or month)		
0–1/month	2382	1.311 (1.069–1.608)
2–4/month	1364	**1.458 (1.133–1.876)**
≥2/week	1161	1.223 (0.941–1.589)
Lifetime cigarette smoking		
No	2893	**1.376 (1.141–1.658)**
Yes	2014	1.255 (1.030–1.529)
Prediabetes or diabetes^†^		
No	3914	1.228 (1.053–1.433)
Yes	993	**1.677 (1.264–2.227)**
Prehypertension or hypertension^‡^		
No	2986	1.279 (1.069–1.530)
Yes	1921	**1.377 (1.120–1.693)**
Obesity^§^		
No	3484	1.300 (1.103–1.533)
Yes	1423	**1.322 (1.041–1.679)**

Periodontitis was defined as community periodontal index 3–4.

*Odds Ratio of dietary vitamin C defined by EAR (Inadequate [<75mg/day] against the reference, adequate [≥75mg/day]) was adjusted for age, sex, income, tooth brushing, use of floss, dental visit, alcohol drinking, smoking, diabetes, hypercholesterolemia, hypertension, and obesity except the subgroup.

^†^Prediabetes or diabetes: fasting blood sugar ≥ 100 mg/dl or on anti-diabetic medicine or insulin injection or on diagnosis by doctor.

^‡^Prehypertension or hypertension: systolic blood pressure (SBP) ≥120 mmHg, diastolic blood pressure (DBP) ≥80mmHg or anti-hypertensive medicine.

^§^Obesity: Body Mass Index (BMI) ≥25 m^2^/kg.

Bold denotes highlighted association with odds ratio more than 1.325 (non-stratified odds ratio in 30–49 years).

## Discussion

Our data showed that the EAR-defined vit C intake was associated with periodontitis after controlling for potential confounders among Korean adults. Our data supports previous studies which found an inverse association between dietary vit C intake and periodontitis [15, 20]. It was speculated that our data also supported other serum or plasma vit c studies which showed a significant inverse association with periodontitis indirectly [11, 14, 16, 17, 19, 30]. Though some clinical studies failed to show effects of oral administration of additional vit C on reducing periodontitis [31–33], they demonstrated that excessive vit C does not have therapeutic benefit for periodontitis while inadequate intake of vit C could be harmful for periodontitis. To the best of our knowledge, this study is the first evidence that applied EAR as daily intake guideline for evaluating the association between dietary vit C intake and periodontitis.

The major strengths of this study are three-fold. Firstly, our data is a representative national data with a large sample size and information of various potential confounders, enabling multivariable analysis and stratified analysis. Secondly, the individual weighted factors were applied by using complex sample analysis. In multistage sampling design, when certain subgroups of the population are oversampled, the estimated association may be biased, and application of weight by complex sample analysis is recommended to attenuate this problem [34]. Finally, we applied the EAR to define dietary vit C intake. Moreover, we additionally applied quartile values and RNI for dietary vit C intake to evaluate a biological-gradient relationship and provide sensitive analysis of the link.

Our data showed that the association between dietary vit C intake and periodontitis was statistically significant among adults aged 30 to 49 years. Periodontitis is a chronic inflammatory disease with multiple risk factors. In young adults aged below 30 years, the prevalence of periodontitis is low, possibly due to the insufficient duration of time exposed to develop periodontitis. In contrast, older adults aged over 50 could have had more chance of multiple tooth extraction due to periodontitis than younger adults. Hence, the effect of inadequate dietary vit C intake on periodontitis could be masked and highlighted only among those aged 30 to 49 years. Further studies are needed to validate our finding that the link between dietary vit C intake and periodontitis is highlighted in those aged 30 to 49 years.

Our data did not show an association between dietary vit C intake and periodontitis among elders, which is different from a Japanese study conducted among elders [16]. It should be considered that the Japanese study was not fully adjusted for systemic factors. Our data showed that the link between vit C and periodontitis was highlighted in females which are in accordance with Dutch study [13]. Males generally have more periodontitis than females [35]. Hence, it is speculated that the effect of vit C on periodontitis could be masked in males due to other risk factors for periodontitis.

Hyperglycemia in diabetics can lead to development of oxidative stress [36]. Since periodontitis is also associated with oxidative stress [9], the scavenging of ROS by antioxidants such as ascorbic acid may be important for the prevention of periodontitis in diabetics and hence, vit C in diabetic adults could mean interdependent effect on developing periodontitis.

In the stratified analysis, the link between vit C and periodontitis was highlighted among those with low income, less 4 times drinking frequency per month, non-smokers, diabetes including pre-diabetes, hypertension including pre-hypertension, and obese individuals. It was reported that the proportion of Korean adults who does not had proper dental treatment was 24.5% for the highest income while those of the lowest, middle-low, middle-high were 34.3, 30.3 and 31.4% [37]. Since alcohol drinking [38,39], smoking [40] and hypertension [41] could be risk factors to periodontitis, the link between vit C deficiency and periodontitis could be masked among heavy drinkers, smokers and hypertensive. On the other hand, the link between vit-C and periodontitis was highlighted among diabetic individuals. Obesity and diabetes mellitus are associated with modulation of immune activity which results in higher susceptibility to infectious diseases such as periodontitis [42,43]. Hence, when there is inadequate intake of vit C, those with diabetes would be more sensitive to oxidative stress for developing periodontitis.

Some biological mechanisms have been suggested to explain how vit C could influence periodontitis. First, vit C is an intracellular antioxidant which scavenges free radicals and protect cells from oxidative stress [8]. Second, vit C functions as an essential co-factor for the hydroxylation of proline and lysine which is vital to collagen biosynthesis in connective tissue [25]. Last but not least, an in vitro study showed that vit C induced the osteogenic differentiation of periodontal ligament progenitor cells through ERK/MARK pathway [44]. Therefore, vit C could possibly stimulate stem cells to differentiate and enhance skeletomuscular development.

Our data has some limitations. Firstly, the cross-sectional study design could not infer the causal relationship between vit C and periodontitis. Thus our data require a longitudinal study for the incidence of periodontitis to infer the causality. Secondly, applying CPI for evaluating periodontitis might underestimate the periodontal prevalence by 50% or more due to partial mouth recordings protocols so there might be misclassification of periodontitis [45]. Thus, full mouth periodontal examination using CPI or clinical attachment loss (CAL) could have provided a more valid periodontitis classification. Thirdly, vit C intake by 24-hour recall method could be different from the actual dietary intake. In order to adjust for day-to-day variations, researchers are encouraged to average multiple 24-hour recall or use statistical approaching method [46,47]. Finally, this investigation did not consider seasonal variation in food intake, nutritional supplement, and number of teeth, respectively. Notwithstanding these limitations, our data is valid enough to address the association between vit C and periodontitis among Korean adults.

## Conclusions

Our data showed that dietary vit C intake was independently associated with periodontitis among Korean adults. The link was in a biological-gradient relationship and highlighted in adults aged 30 to 49 years. Hence, adequate intake of dietary vitamin C could be substantially important on the promotion of periodontal health among Korean adults.

## Supporting information

S1 STROBE_Checklist (DOC)

## Abbreviations

vit C: vitamin C
EAR: estimated average requirement
RNI: Recommended Nutrient Intake
